# Assessing Development Assistance for Mental Health in Developing Countries: 2007–2013

**DOI:** 10.1371/journal.pmed.1001834

**Published:** 2015-06-02

**Authors:** Barnabas J. Gilbert, Vikram Patel, Paul E. Farmer, Chunling Lu

**Affiliations:** 1 Graduate School of Arts and Sciences, Harvard University, Cambridge, Massachusetts, United States of America; 2 Department for Population Health, Centre for Global Mental Health, London School of Hygiene and Tropical Medicine, London, United Kingdom; 3 Centre for the Control of Chronic Conditions, Public Health Foundation of India, Gurgaon, India; 4 Department of Global Health and Social Medicine, Harvard Medical School, Boston, Massachusetts, United States of America; 5 Division of Global Health Equity, Brigham and Women’s Hospital, Department of Global Health and Social Medicine, Harvard Medical School, Boston, Massachusetts, United States of America

## Abstract

Chunling Lu and colleagues investigate how international aid spent on mental health projects has changed between 2007 and 2013.

**Summary Points**

- Mental disorders are a leading cause of the global burden of disease, and the provision of mental health services in developing countries remains very limited and far from equitable.
- Using the Creditor Reporting System, we estimate the amounts and patterns of development assistance for global mental health (DAMH) between 2007 and 2013. This allows us to examine how well international donors have responded to calls by global mental health advocates to scale up evidence-based services.
- Although DAMH did increase between 2007 and 2013, it remains low both in absolute terms and as a proportion of total development assistance for health (DAH). The average annual DAMH between 2007 and 2013 was US$133.57 million, and the proportion of DAH attributed to mental health is less than 1%.
- Approximately 48% of total DAMH was for humanitarian assistance, education, and civil services. More annual DAMH was channelled into the nonpublic sector than the public sector.
- Despite an expanding body of evidence suggesting that sustainable mental health care can be effectively integrated into existing health systems at relatively low cost, mental health has not received significant development assistance.

## The Need for Investment in Global Mental Health

Mental and substance-use disorders account for approximately 8% of the global burden of disease, afflicting as many as 700 million people worldwide [1,2]. The most recent estimates rank mental and substance-use disorders third in the leading global causes of disability-adjusted life years, accounting for 23% of all years lived with disability—more than cardiovascular diseases or cancer [1,2].

Figures such as these tend to underestimate the true burden of disease, because of the universal complexity of diagnosing and reporting mental illness and a narrow definition of disease burden that fails to incorporate impact on families or society [3,4]. The economic burden posed by mental disorders on society is immense, with global costs estimated at US$2.5 trillion in 2010 and forecast to reach US$6.0 trillion by 2010 [5]. Mental health is also intrinsically linked to a range of Millennium Development Goals, including gender equality, improving maternal and child health, and the outcome of HIV/AIDS [3,6].

Globally, the provision of mental health services remains very limited and far from equitable. The World Health Organization (WHO) reports that 75% of people with mental disorders live in low- or middle-income countries and the majority do not have access to any kind of care, despite evidence to support a range of cost-effective pharmacological, psychological, and social interventions [7,8]. The 2011 WHO Mental Health Atlas reports that, on average, low-income countries devote 0.5% of their health budget to mental health, compared to 1.9% in lower-middle-income countries, 2.4% in upper-middle-income countries, and 5.1% in high-income countries [9]. Whilst the global median mental health expenditure was US$1.63 per capita per annum, there was a large variation between income groups, ranging from US$0.20 in low-income countries to US$44.80 in high-income countries [9].

In 2007, *The Lancet* published a series on global mental health [10], which was followed by two series in *PLOS Medicine* in 2009 and 2012, respectively [11,12]. Together, these articles called for scaling up of evidence-based services for mental disorders in low- and middle-income countries. Whilst such interventions require sufficient and sustainable funding, low levels of national funding for mental health in developing countries begs the question of what international donor organizations are doing to supplement these resources. Here, we provide estimates and patterns of development assistance for mental health (i.e., aid spent on projects that were related to promoting mental health and preventing and treating mental disorders) since 2007.

## Estimating Development Assistance for Mental Health (DAMH) between 2007 and 2013

Following the methodology used in previous studies tracking development assistance for health (DAH) [13–18], we used the aid activities database from the Creditor Reporting System of the Organisation for Economic Co-operation and Development’s Development Assistance Committee (DAC) to trace DAMH between 2007 and 2013. The estimation methods used are similar to these previous studies [13–18], with special considerations for mental health described below.

### Data Sources

The Creditor Reporting System database is publicly accessible and provides information on aid activities [19] reported directly by the governments of the 26 members of the DAC (mandatory), multilateral organizations (such as the United Nations and World Bank), global health initiatives (such as the Global Fund to Fight AIDS, Tuberculosis and Malaria), non-DAC countries (such as the United Arab Emirates), and private donors (such as the Bill & Melinda Gates Foundation) [20]. The Creditor Reporting System is considered the most comprehensive and authoritative data source on development assistance projects. The data used in the present study were obtained in July 2014 and March 2015, with projects funded by 55 donors and implemented in 157 developing countries.

### Identifying Mental Health Projects and Measuring DAMH

We defined DAMH as aid spent on projects whose primary purpose was promoting mental health or preventing or treating mental and substance-use disorders. The Creditor Reporting System data do not have a variable to indicate mental health projects but have three variables (project title, short description of a project, and long description of a project) that enabled us to identify such projects via a list of keywords (S1 Table). The keywords were constructed based on the diagnoses for mental and behavioural disorders listed in the Tenth Revision of the International Classification of Diseases (ICD-10) [21]. Our keyword search was conducted for projects listed in the sectors presented in Table 1.

**Table 1:** Sectors for identifying mental health projects: Frequency and examples.

	Frequency and Percentage of Total Projects N = 5,212	Example
***Education***		
Basic Education	198 (3.80%)	Education and training for students with mental disabilities
Secondary Education	21 (0.40%)	Training and psychological support
Post-secondary Education	72 (1.38%)	Psychological intervention and development project for poor university students
Education, Level Unspecified	95 (1.82%)	Strengthening mental health in teenagers
***Health***		
Health, General	1,688 (32.39%)	Psychosocial aid in rural Afghanistan—support for post-traumatized war victims and socially marginalized people
Basic Health	791 (15.18%)	Supervision services of the rehabilitation and construction of the psychiatric women’s hospital in Bethlehem
Population and Reproductive Health	161 (3.09%)	Providing comprehensive community-based mental services
***Government and Civil Services***		
Government and Civil Society	641 (12.30%)	Review of the national prison system and the mental health law
Conflict, Peace, and Security	207 (3.97%)	Trauma development and peacebuilding: towards an integrated psychological approach
***Other Social Infrastructure and Services***		
Other Social Infrastructure and Services	764 (14.66%)	Construction of a youth and mental health education and counselling centre
***Humanitarian aid***		
Emergency Response	518 (9.94%)	Community psychosocial and mental services—West Bank and Gaza
Reconstruction Relief and Rehabilitation	42 (0.81%)	Integrating mental health into the primary health care system of Afghanistan
Disaster Prevention and Preparedness	14 (0.27%)	Mental health preparedness in public health emergency settings

Following the practices in previous studies [13–18], we used actual disbursements (grants), rather than commitments, to estimate donors’ contributions to mental health projects between 2007 and 2013. The time frame allows us both to track changes in DAMH since 2007 and to avoid the issue of missing disbursement data: the completeness of disbursement data has remained at almost 100% since 2007 [22].

We aggregated yearly disbursements for all identified mental health projects and tracked trends in DAMH by total, donors, recipients, sectors, and channels of delivery. As per the 2010 DAH study [14], we defined DAMH as going into the “public sector” if a project’s channel of delivery was either public sector or a public—private partnership. Disbursements going to governments of donor countries or other countries are also included in the “public sector” in the Creditor Reporting System. Disbursements going to nongovernmental organizations, civil society, multilateral organizations, or others were defined as disbursements to the “nonpublic sector.” We present names and codes of channels of delivery under these two categories in S2 Table. Approximately 36% of mental health projects lacked information on the channel of delivery; on the basis of the 2010 study [14], we assumed they were directed into the public sector. Further details on data and estimation can be found in the S1 Text.

## Absolute and Relative DAMH

**Fig 1:**
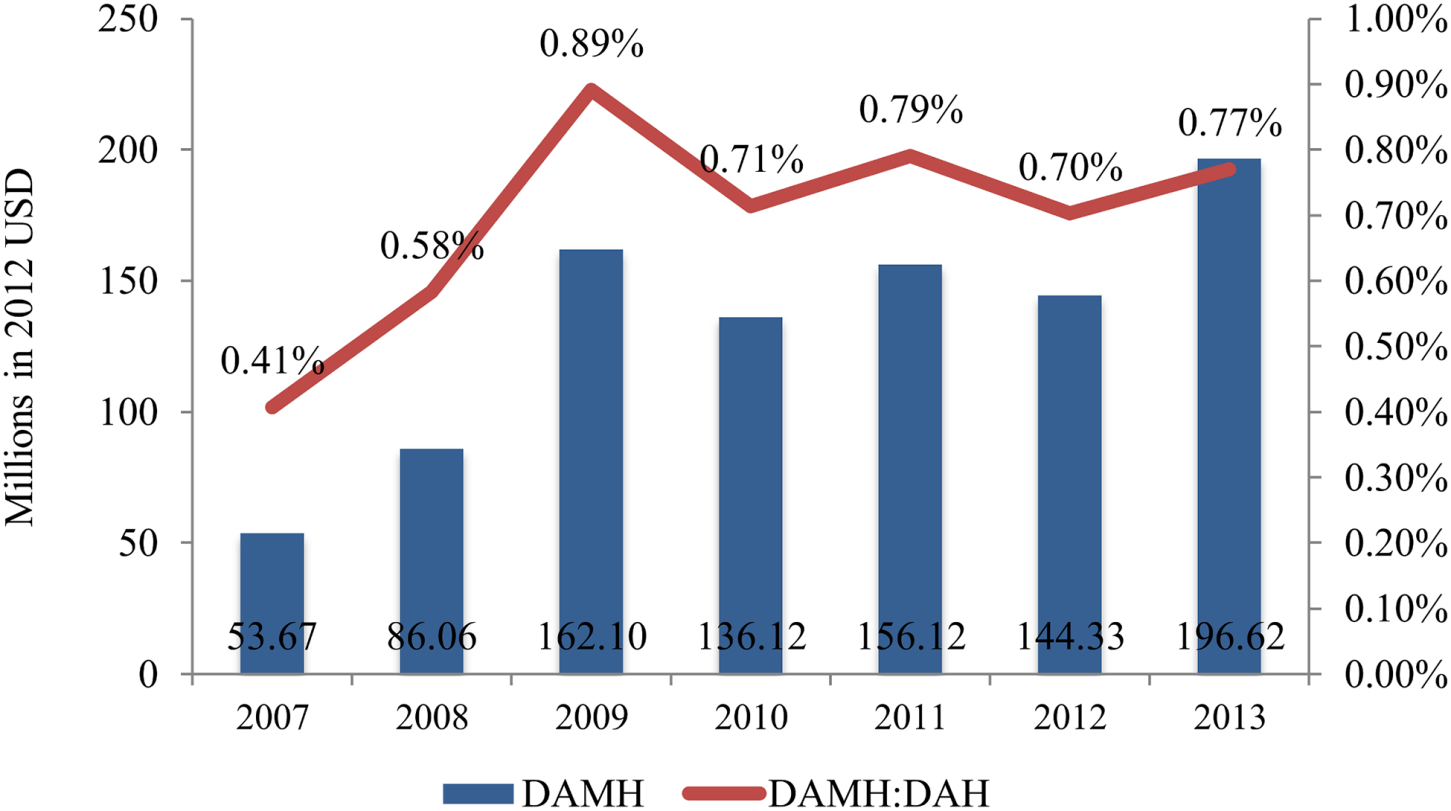
Trends in annual DAMH and its proportion of DAH, 2007–2013.

Between 2007 and 2013, total DAMH had a mean of US$133.57 million, increasing from US$53.67 million in 2007 to a peak of US$196.62 million in 2013 (Fig 1). Annual DAMH has increased by more than three times since 2007.

As a percentage of total DAH, DAMH ranges from 0.41% in 2007 to 0.77% in 2013, with a mean of 0.69% (Fig 1). The highest percentage is in 2009 (0.89%). The proportion of DAMH in total DAH remains less than 1% during the period, despite a 3-fold increase in its absolute value.

## DAMH Donors and Recipients

Among the 55 donors who reported to the Creditor Reporting System, 38 disbursed aid to mental health projects: 28 bilateral government donors, nine multilateral organizations, and one private organization (Bill & Melinda Gates Foundation). Bilateral donors contributed the largest cumulative disbursements to DAMH during the period (US$497.05 million), followed by multilateral donors (US$437.29 million). The Bill & Melinda Gates Foundation donated US$0.67 million to Haiti in 2010 to provide emotional and psychological support to children affected by the earthquake. Annual disbursement by donor type shows that, before 2011, bilateral donors disbursed more aid to DAMH than multilateral donors. Multilateral donors have been increasing their contribution to DAMH steadily since 2007 and took the lead between 2011 and 2013 (S2 Fig). Of all donors, the largest single donor was the WHO, with a cumulative DAMH disbursement between 2007 and 2013 totalling US$211.04 million, followed by the European Union institutions (US$152.85 million) and the governments of the United States (US$88.14 million), Norway (US$72.19 million), and Germany (US$62.75 million) (S3 Fig). The top ten donors contributed approximately 83.5% of total DAMH during the period.

Among 157 recipient countries, 148 received DAMH during the period. Lower-middle-income countries received the largest cumulative DAMH funding between 2007 and 2013 (US$495.87 million), followed by low-income countries (US$301.62 million) and upper-middle-income countries (US$137.53 million) (S4 Fig). Based on population data from the UN’s “World Population Prospects” [23], the estimated DAMH per capita in 2011 was US$0.05 for low-income countries, US$0.02 for lower-middle-income countries, and US$0.03 for upper-middle-income countries. For regions, the DAMH per capita in 2011 is US$0.07 in Africa, $0.04 in Europe and Oceania, US$0.03 in America, and US$0.02 in Asia. Among 148 recipient countries, countries or states involved in wars during the period, such as the West Bank and Gaza Strip, Senegal, the Democratic Republic of the Congo, Afghanistan, and Sri Lanka, received the largest cumulative DAMH (S5 Fig).

## Sectors Receiving DAMH

**Fig 2:**
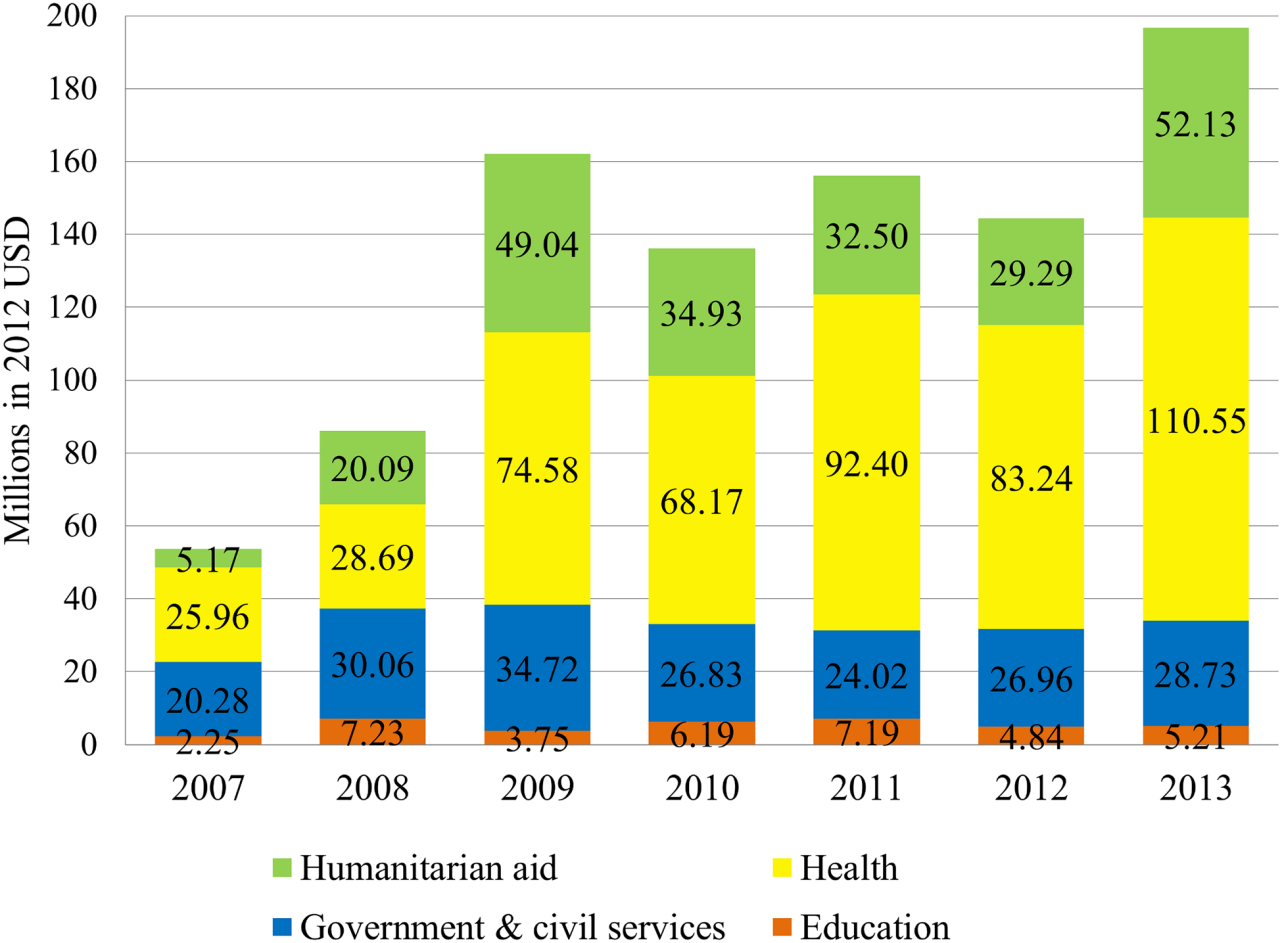
Annual DAMH by sector, 2007–2013.

The health sector received the largest cumulative DAMH funding over the period (US$483.60 million), followed by humanitarian aid (US$223.15 million), government and civil services (US$191.61 million), and education (US$36.66 million). The annual DAMH by sector is presented in Fig 2. Before 2009, most DAMH went to civil services, humanitarian assistance, and education. Since 2009, DAMH to the health sector increased substantially but remains less than 60% of total DAMH.

**Fig 3:**
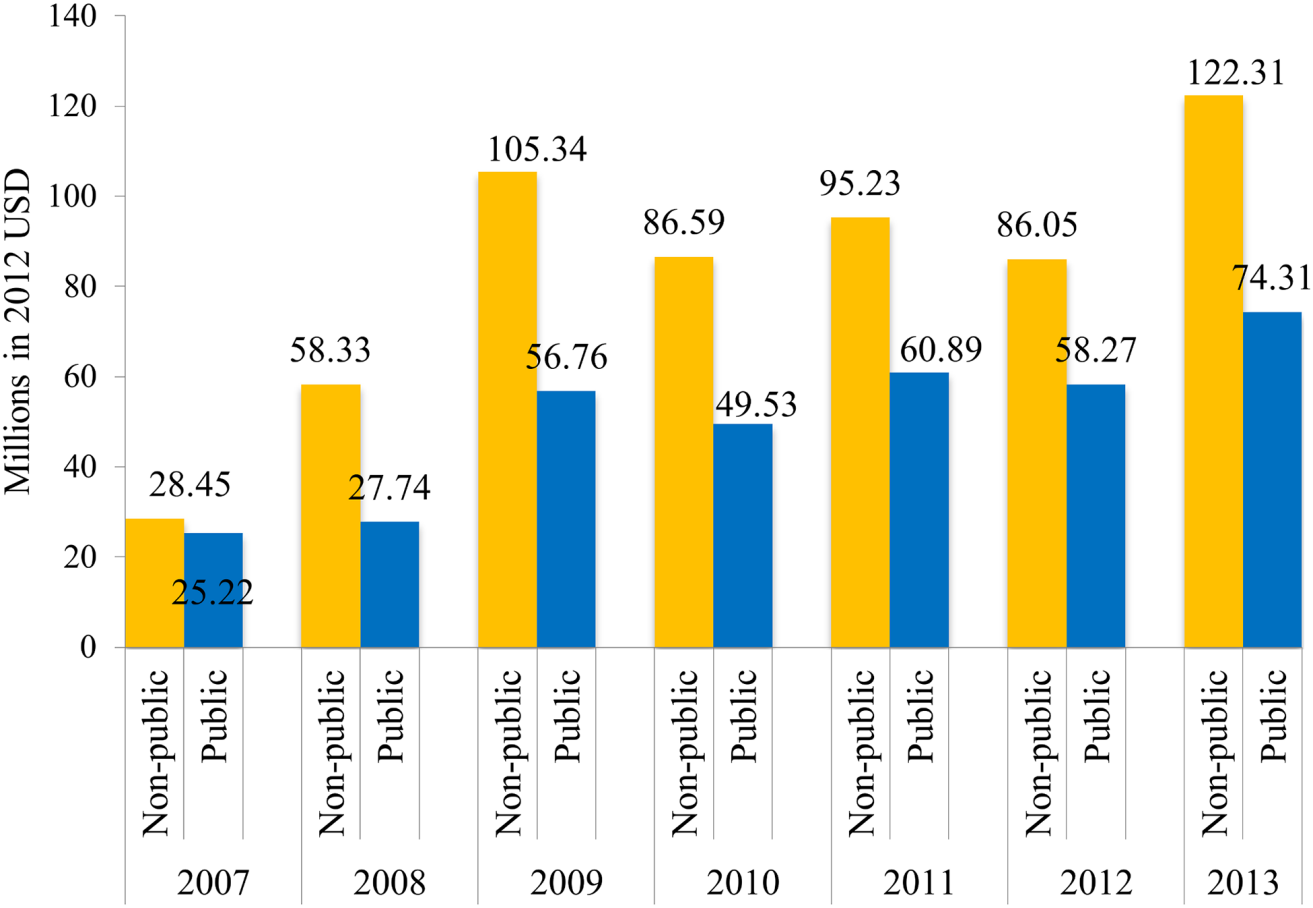
Annual DAMH by channel of delivery (assuming funding for projects with missing channels goes to public sector), 2007–2013.

In terms of channel of delivery, from 2007 to 2013, the nonpublic sector received a cumulative DAMH of US$582.30 million, and the public sector received a cumulative DAMH of US$114.65 million. The cumulative value of the DAMH projects with a missing channel of delivery is US$238.06 million. Assuming that disbursements of projects without information for channel of delivery went to the public sector, Fig 3 shows that the annual DAMH going to the nonpublic sector is consistently higher than that to the public sector.

## Discussion

Using the Creditor Reporting System data and tracking 38 donors’ aid disbursements to mental health projects implemented in 148 developing countries, we found that, despite an increase since 2007, DAMH remains low both in absolute terms and as a proportion of DAH between 2007 and 2013. During the period, mental health projects in nonhealth sectors accounted for approximately 48% of total DAMH, and more annual DAMH was channelled into the nonpublic sector than the public sector. These findings suggest that integrating mental health care into the public health sector has not received significant development assistance.

**Fig 4:**
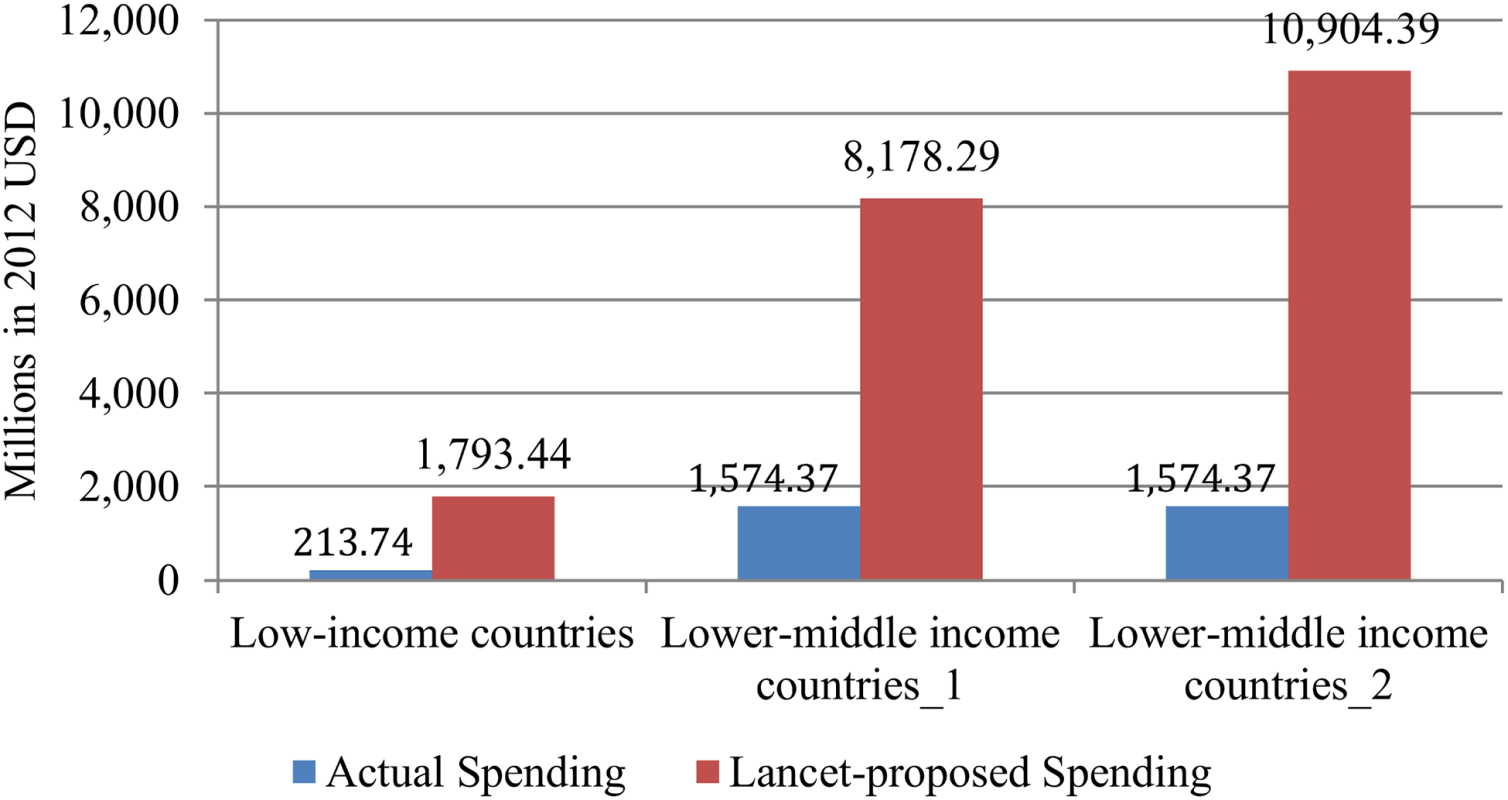
Financial gap between actual spending and spending proposed by the Lancet Global Mental Health Group (LGMHG). LIC: low-income country; LMIC: lower-middle-income country; LMIC_1: based on US$3 per capita LGMHG estimation; LMIC_2: based on US$4 per capita LGMHG estimation.

The 2011 WHO Mental Health Atlas estimated mental health expenditure in 2011 as US$0.20 per capita in low-income countries and US$0.59 per capita in lower-middle-income countries [9]. Together with DAMH in 2011, total spending on mental health per capita equates to approximately US$0.25 in low-income countries and $0.61 in lower-middle-income countries. These values lie far below estimates for the cost of scaling up a basic mental health care package of US$2 per capita per year in low-income countries and US$3–US$4 in lower-middle-income countries [24]. After converting all estimates into 2012 US dollars, the shortfall is US$1.88 per person (US$1,580 million in total) in low-income countries and US$2.62–US$3.70 per person (US$6,600–US$9,330 million in total) in lower-middle-income countries (Fig 4).

We want to point out that, because of unavailable data, our estimates did not include aid for mental health from many private nongovernmental organizations. Data for bilateral aid from emerging economies such as China or Brazil to other developing countries are also not available. It has been demonstrated that approximately 22% of health aid is from nongovernmental organizations [25]. Information for allocating nongovernmental organizations’ aid for mental health is not available. If their disbursements to mental health mirrored the donors in the present study, our principal finding of low-level estimates for DAMH will remain unchanged.

The past decade has witnessed a dramatic increase in DAH to support developing countries in meeting the health-related Millennium Development Goals [25]. DAH plays an essential role in financing the health sector of the least-resourced countries. According to the WHO’s Global Health Expenditure Database, health aid accounted for between 20% and 56% of total health expenditure in 24 sub-Saharan countries in 2010 [26]. While low-income countries are not able to finance mental health care by their own resources, increasing DAMH could substantially improve the financing of mental health in these countries and support the integration of low-cost interventions into existing health systems. Unfortunately, the increase in DAMH after 2007 is simply too small. The less than 1% of DAH channelled into mental health suggests that mental illness is indeed among the “most neglected of neglected diseases” [27].

A growing body of evidence suggests that effective interventions could be integrated into existing health systems at relatively low cost in developing countries [8,24,28,29]. It is estimated that every US$1 million invested in an effective treatment package for mental health would generate 350–700 million extra healthy life years [28]. Despite this evidence on the effectiveness and affordability of mental health interventions in developing countries, our findings show that mental health remains a low priority in the agendas of funders of development assistance for health. While many middle-income countries like India, China and Brazil, which are not reliant on DAH, have ramped up their funding for scaling up mental health services, there is little sign of this evidence being put into action in low-income countries, which are reliant on DAH. While there are several well-described reasons that may explain the apparent lack of enthusiasm in investing in mental health [30], we believe that this generally reflects the stigmatized and misinformed attitudes towards mental health problems that pervade the DAH community—for example, the misconceptions that mental disorders are not problems of the poor, that mental health problems do not kill, and that cost-effective treatments are not available in low-resource settings.

The pressing message of this study is that, to prioritize the actions proposed by global mental health advocates, DAMH must increase, with emphasis on integrating a basic mental health package into the public health sector. In developing countries, the public health sector plays a key role in delivering basic health care services to the most vulnerable populations through community-based approaches. Evidence from countries shows that a basic mental health treatment package can be effectively implemented via task-shifting programs that engage community health workers in care delivery [30–32]. Such programs should be better documented and communicated to donors and policy makers alike. DAMH should be used to strengthen the capacity of the public health sector for building an effective and sustainable mental health care system beyond the period of assistance [33].

The need for specified mental health funding should be clearly advocated to development aid agencies in well-resourced countries, by raising awareness of the burden of mental illness, its far-reaching socioeconomic impact and the benefits of treatment, and the current deficit in mental health financing. In the short term, DAMH increases should be drawn from governmental, multilateral, and nongovernmental sources, and leading donors should specify a budgetary allocation for mental health. Transition will be needed in the medium-to-long term to encourage investment by the governments of developing countries, who must ultimately hold responsibility for mental health at the population level. An important step in this direction will be to incorporate explicit goals for mental health in the UN’s emerging Sustainable Development Goals [34]; targets for universal health coverage, for example, should explicitly assess coverage for mental disorders.

## Supporting Information

S1 Fig Percentage of country-unspecified DAMH in total DAMH, 2007–2013. (DOCX)

S2 Fig Annual DAMH by donor type, 2007–2013. (DOCX)

S3 Fig Top ten donors for cumulative DAMH, 2007–2013. (DOCX)

S4 Fig Annual DAMH by income group, 2007–2013. LIC: low-income country; LMIC: lower-middle-income country; UMIC: upper-middle-income country. (DOCX)

S5 Fig Top ten recipients with the largest cumulative DAMH, 2007–2013. (DOCX)

S1 Table Keywords used to search for mental health projects in the Creditor Reporting System, 2007–2013. (DOCX)

S2 Table Channels in public and nonpublic sectors. (DOCX)

S1 Text The Creditor Reporting System database; Identifying mental health related projects via keywords search; Allocating regional or global funds into recipient countries. (DOCX)

## Abbreviations

DAC: Development Assistance Committee
DAH: development assistance for health
DAMH: development assistance for mental health
ICD-10: Tenth Revision of the International Classification of Diseases
LGMHG: Lancet Global Mental Health Group
WHO: World Health Organization
