# Supplementary material for: Assessing Development Assistance for Mental Health in Developing Countries: 2007–2013
Source: PLoS Med. 2015 Jun 2;12(6):e1001834. doi: 10.1371/journal.pmed.1001834 (PMC4452770; doi:10.1371/journal.pmed.1001834)
Supplement: S1 Fig — (DOCX) [file pmed.1001834.s001.docx]

**S1 Figure. Percentage of country-unspecified DAMH in total DAMH, 2007-2013**
