# Supplementary material for: Assessing Development Assistance for Mental Health in Developing Countries: 2007–2013
Source: PLoS Med. 2015 Jun 2;12(6):e1001834. doi: 10.1371/journal.pmed.1001834 (PMC4452770; doi:10.1371/journal.pmed.1001834)
Supplement: S4 Fig — (DOCX) [file pmed.1001834.s004.docx]

**S4 Figure. Annual DAMH by income group, 2007-2013**

LIC: low-income country; LMIC: lower-middle-income country; UMIC: upper-middle-income country.
