# Supplementary material for: Assessing Development Assistance for Mental Health in Developing Countries: 2007–2013
Source: PLoS Med. 2015 Jun 2;12(6):e1001834. doi: 10.1371/journal.pmed.1001834 (PMC4452770; doi:10.1371/journal.pmed.1001834)
Supplement: S1 Table — (DOCX) [file pmed.1001834.s006.docx]

**S1 Table. Keywords used to search for mental health projects in the Creditor Reporting System, 2007-2013**

| Addiction | Cognitive impairment | Hallucination | Psychia- |
| --- | --- | --- | --- |
| Affective | Delirium | Hyperactivity | Psycho- |
| Alcohol | Delusion | Hypochondriasis | Schizo- |
| Antidepressant | Dementia | Insomnia | Self-harm |
| Antipsychotic | Dependency | Learning disability | Sleep disorder |
| Anxiety | Depress | Mania | Somatoform |
| Anxiolytic | Dyslexia | Mental | Trauma |
| Autism | Eating disorder | Mood | Substance abuse |
| Behavioural therapy | Electroconvulsive therapy | Personality disorder |  |
| Bipolar | Family therapy | Phobia |  |
