# Supplementary material for: Assessing Development Assistance for Mental Health in Developing Countries: 2007–2013
Source: PLoS Med. 2015 Jun 2;12(6):e1001834. doi: 10.1371/journal.pmed.1001834 (PMC4452770; doi:10.1371/journal.pmed.1001834)
Supplement: S2 Table — (DOCX) [file pmed.1001834.s007.docx]

**S2 Table. Channels in public and non-public sectors**

|  | Channel code | Channel name |
| --- | --- | --- |
| Public | 10000 | PUBLIC SECTOR (donor, recipient, other) |
|  | 11000 | Donor government |
|  | 12000 | Recipient government |
|  | 13000 | Third Country Government (Delegated co-operation) |
|  | 30000 | PUBLIC-PRIVATE PARTNERSHIPS |
| Non-public | 20000 | NON-GOVERNMENTAL ORGANISATIONS (NGOs) AND CIVIL SOCIETY |
|  | 21000 | INTERNATIONAL NGOs |
|  | 21016 | International Committee of the Red Cross |
|  | 21018 | International Federation of Red Cross and Red Crescent Societies |
|  | 21029 | Doctors Without Borders |
|  | 22000 | NATIONAL NGOs |
|  | 23000 | LOCAL/REGIONAL NGOs |
|  | 40000 | MULTILATERAL INSTITUTIONS |
|  | 41000 | UNITED NATIONS AGENCIES, FUNDS AND COMMISSIONS |
|  | 41114 | United Nations Development Programme |
|  | 41119 | United Nations Population Fund |
|  | 41121 | United Nations Office of the United Nations High Commissioner for Refugees |
|  | 41122 | Contribution to implementing the recommendations of the UNICEF study on gender-based violence |
|  | 41126 | United Nations Mine Action Service |
|  | 41128 | United Nations Office on Drugs and Crime |
|  | 41130 | United Nations Relief and Works Agency for Palestine Refugees in the Near East |
|  | 41140 | World Food Programme |
|  | 41143 | World Health Organisation - core voluntary contributions account |
|  | 41301 | Food and Agricultural Organisation |
|  | 41305 | United Nations |
|  | 41307 | World Health Organisation - assessed contributions |
|  | 42000 | EUROPEAN UNION INSTITUTIONS |
|  | 42001 | European Commission - Development Share of Budget |
|  | 47000 | OTHER MULTILATERAL INSTITUTIONS |
|  | 47046 | International Organisation of the Francophonie |
|  | 47066 | International Organisation for Migration |
|  | 47083 | Pan-American Health Organisation |
|  | 50000 | OTHER |
|  | 51000 | University, college or other teaching institution, research institute or think-tank |
