# Supplementary material for: Assessing Development Assistance for Mental Health in Developing Countries: 2007–2013
Source: PLoS Med. 2015 Jun 2;12(6):e1001834. doi: 10.1371/journal.pmed.1001834 (PMC4452770; doi:10.1371/journal.pmed.1001834)
Supplement: S1 Text — (DOCX) [file pmed.1001834.s008.docx]

**S1 Text**

1. **The Creditor Reporting System database**

Donors in the Creditor Reporting System are classified as bilateral and multilateral. Bilateral aid usually refers to the assistance that comes from a country’s government, and multilateral aid usually refers to assistance from international organizations such as the World Bank, United Nations, etc.

One advantage of the Creditor Reporting System data is that it avoids double counting by classifying a donor country’s government contributions to multilateral organizations as multilateral aid if the donor has no control over the money. If a donor country contracts a multilateral organization to implement a project, then the donor’s contribution to the project is considered as bilateral.^1^

The Creditor Reporting System provides project-level data, with descriptive and financial information linked to each project such as a project’s title, donor(s), recipient(s), implementation year, annual commitments and disbursements, purpose, etc. Each project is assigned to one of 36 sectors (e.g. health, education, agriculture) based on its purpose. It updates its website every three months to release newly revised data.^1^

1. **Identifying mental health related projects via keywords search**

The keywords (S1 Table) were translated into 11 languages (English, French, German, Spanish, Danish, Finnish, Italian, Dutch, Norwegian, Portuguese, and Swedish). If a project’s title contained one or more of these keywords, the project was defined as a mental health project. Note that one project may have various activities and not all of them are related to mental health. For example, the Global Fund to Fight AIDS, Tuberculosis and Malaria supported a project aiming to improve economic and psychosocial care for HIV patients. Based on available information in the Creditor Reporting System, we are not able to know the aid attached to each of these activities in a project. Our estimates can thus be considered as upper bounds for DAMH given the donors and recipient countries in this study.

The Creditor Reporting System documents a donor’s annual commitments and disbursements attached to a specific project in a specific year to a specific recipient country. We used actual disbursements, defined as “the placement of resources at the disposal of a recipient country or agency, or in the case of internal development-related expenditures, the outlay of funds by the official sector”,^1^ to calculate the contribution to DAMH. We only included grants and excluded loan from the estimation.

Unlike previous studies which focused on projects in health sectors only,^2, 3^ we applied the keyword search through projects in health sectors (general health, basic health, and reproductive health) as well as other sectors as listed in Table 1. The search was conducted for all projects in these sectors reported between 2007 and 2013 and identified 4,087 projects targeting mental health during the period.

All disbursements were reported in 2012 USD. All analyses were conducted in Stata (version 12.0).

1. **Allocating regional or global funds into recipient countries**

In the Creditor Reporting System data, some donors reported disbursements only at the regional level or simply labelled the funds as going to “developing countries”. Our study shows that the country-unspecified funding accounts for a substantial proportion of total DAMH (S1 Figure). In 2012, for example, approximately 36% of DAMH was not specified by recipient countries or regions. Without accounting for these regional or global funds, the estimates at the country or regional level will be substantially underestimated.

We allocated the annual regional or global DAMH to each country based on a country’s share in total DAMH in that year. Details are summarized in the following steps.

Step 1 Summing country-specific disbursements across all countries in region g, sector s, and year t. For example, in year 2010, if DAMH was disbursed to health sectors in 50 countries in Africa, we obtained the sum of the country-specific DAMH in the health sector of Africa in 2010 (DAMH_Africa/2010/health_).

Step 2 For each country in a region, aggregating DAMH a country received in sector s and year t. For example, for Rwanda, we aggregated DAMH going to its health sector in 2010 (DAMH_Rwanda/2010/health_).

Step 3 Obtaining a country’s proportion of its received DAMH in sector s and year t in the region. In Rwanda example, in year 2010, its proportion in total DAMH in Africa = (DAMH_Rwanda/2010/health_) / (DAMH_Africa/2010/health_).

Step 4 Multiplying each country’s proportion in year t to country-unspecified disbursements in the region in year t. If in year 2010, $Z was labelled as “Africa, regional”, the amount allocated from $Z to Rwanda is

DAMH_Rwanda/2010/health/allo_ = [(DAMH_Rwanda/2010/health_) / (DAMH_Africa/2010/health_)]*Z.

Step 5 Adding the product derived in step 4 to a country’s received DAMH in sector s and year t. In the Rwanda example, its total DAMH in the health sector in 2010 = DAMH_Rwanda/2010/health/allo_ + DAMH_Rwanda/2010/health_.

**References**

1. OECD. Technical Guide to terms and data in the Creditor Reporting System (CRS) Aid Activities database. Available from: <http://www.oecd.org/dac/stats/crsguide.htm>
2. Ravishankar N, Gubbins P, Cooley RJ, Leach-Kemon K, Michaud CM, Jamison DT, et al. Financing global health: tracking development assistance for health from 1990 to 2007. Lancet. 2009;373(9681):2113-2124.
3. Powell-Jackson, T, Borghi J, Mueller DH, Patouillard E, Mills A. Countdown to 2015: tracking donor assistance to maternal, newborn and child health. Lancet. 2006;368(9541):1077–1087.
